# Jianpi-yangwei decoction inhibits DNA damage repair in the drug resistance of gastric cancer by reducing FEN1 expression

**DOI:** 10.1186/s12906-020-02983-8

**Published:** 2020-06-26

**Authors:** Wenjie Huang, Huijuan Tang, Fang Wen, Xiaona Lu, Qingpei Li, Peng Shu

**Affiliations:** 1 Oncology Department, Affiliated Hospital of Nanjing University of Chinese Medicine, 155 Hanzhong Road, Jiangsu province Nanjing, 210029 China; 2 Department of Clinical and Molecular Sciences, Università Politenica delle Marche, 60126 Ancona, Italy

**Keywords:** Gastric cancer, Jianpi Yangwei decoction, FEN1, DNA damage repair, Drug resistance

## Abstract

**Background:**

Flap Endonuclease 1(FEN1) has been considered as a new tumor marker in recent years and Jianpi Yangwei Decoction (JPYW) is a basic Traditional Chinese Medicine (TCM) for the treatment of gastric cancer. This study aimed to explore the role of FEN1-mediated DNA damage repair in the drug resistance of gastric cancer and the effect of JPYW on it by employing BGC823/5-Fu drug-resistant cell model.

**Methods:**

The DNA repair efficiency of BGC823 and BGC823/5-Fu was compared intracellularly and extracellularly using an extrachromosomal assay system and the reconstituted base excision repair assay. By comparing gene and protein expression and identifying cell survival rates after knockdown or high expression of FEN1, the correlation between FEN1 high expression and 5-Fluorouracil (5-Fu) drug resistance was revealed. The effect of JPYW on DNA damage repair and FEN1 expression was observed by the degree of γ-H2AX phosphorylation in the cells, DNA repair efficiency and enzyme activity, et al.

**Results:**

BGC823/5-Fu had a higher DNA repair efficiency than BGC823(*P* < 0.001), which proved to be both intracellular and extracellular. FEN1 was highly expressed in BGC823/5-Fu regardless of gene level(P < 0.001) or protein level. Furthermore, manipulating FEN1 altered the sensitivity of cancer cells to chemotherapeutic drug 5-Fu. Different concentrations of JPYW were used to investigate the inhibitory effect on the expression of FEN1 and DNA damage repair. JPYW inhibited DNA damage repair both intracellularly and extracellularly: the phosphorylation of γ-H2AX increased, with more DNA damage in the cells; the synthetic 8-oxo dG damage repair was reduced; and the ability of cell lysates to repair DNA damage decreased. The decrease of FEN1 expression in BGC823/5-Fu had a concentration dependent relationship with JYPW. In addition, JPYW inhibited the activity of FEN1 at the enzymatic level, as the amount of cut-off synthetic ^32^p labeled DNA substrates were decreased.

**Conclusion:**

FEN1 was highly expressed in drug-resistance gastric cancer cells BGC823/5-Fu, which leading to BGC823 resistant to (5-Fu) by acting on DNA damage repair. JPYW inhibited DNA damage repair and reversed 5-Fu drug resistance by reducing FEN1 expression and inhibiting FEN1 functional activity.

## Background

Gastric cancer is one of the major causes of tumor-related deaths worldwide, according to the global cancer statistics in 2018, it is the fifth most frequently diagnosed cancer and the third leading cause of cancer deat h[1]. Chemotherapy is the main method of postoperative treatment for gastric cancer, 5-Fluorouracil (5-Fu) and its derivatives are the cornerstones of standard chemotherapy regiments for gastric cancer. However, the emergence of new drugs and new regimens have not effectively improved the survival rate, one of the important reasons is that the presence of multi-drug resistance (MDR) of gastric cancer cells reduce its sensitivity to chemotherapy (especially 5-Fu).

In recent years, DNA damage repair has been found to be a new and crucial mechanism for drug resistance of tumor. Most of the chemotherapy drugs in clinic are used to clear tumor cells by inducing apoptosis through DNA damag e[2] .At the same time, sustained DNA damage may excite the damage repair mechanism of tumor cell DNA, causing rapid emergence of secondary drug resistance to chemotherapy drugs. Thus, the clinical efficacy is greatly reduced. The DNA Base Excision Repair (BER) is one of the most important pathways to repair DNA damag e[3]. Most chemotherapeutic drugs, especially DNA synthesis inhibitors such as alkylating agents, base analogs (like 5-Fu), cause DNA damage repair by BER. Flap Endonuclease 1 (FEN1) is a structure-specific endonuclease. Along with apurinic/apyrimidinic endonuclease 1(APE1), DNA polymerase beta (Pol Beta) and X-ray Repair complementing defective repair in Chinese hamster cells 1(XRCC1), it is an important component of the Long patch BER pathway (LP-BER). High FEN1 expression is often a common feature of active replication cells, which is consistent with the fact that tumor cells, due to their vigorous growth speed, have a higher FEN1 expression than normal cells. Therefore, FEN1 has been considered as a new tumor marker in recent year s[4]. Studies have shown that high expression of FEN1 is associated with drug resistance in breast cancer,[5] lung cancer,[6]cervical cance r[7] and osteosarcom a[8]. And its involvement in tumor drug resistance has been confirmed.

In recent years, research on DNA damage repair mediated tumor drug resistance reversers have been carried out constantly. However, most of the drugs are still in the trial and development stage and have not been put into clinical use yet. Therefore, scholars turn their attention to the researches on multi-target, low-toxic Chinese medicine reversal agents. Our research team has conducted decades of clinical and basic researches on the traditional Chinese medicine Jianpi Yangwei Decoction and proved the excellent efficacy of this decoction in anti gastric cancer drug resistanc e[9–13].

Based on the above theoretical basis and previous studies, further experiments were conducted to explore whether FEN1 mediated DNA damage repair is an important mechanism of gastric cancer resistance to 5-Fu; to research the inhibitory effect of Chinese Medicine Jianpi Yangwei Decoction on FEN1 expression and DNA damage repair; and to determine its efficacy in reversing the resistance of gastric cancer to 5-Fu.

## Methods

### Cell lines

Human gastric cancer cells BGC823 were purchased from ATCC. All cells were cultured at 37 °C in a humidified 5% CO_2_ incubator. BGC823/5-Fu cells were established by a long term continuous exposure of BGC823 cells to 5-Fu in stepwise increase of concentration. The drug RI of BGC823/5-Fu was 31 determined by CCK8 assay.

### Antibody

Antibodies used in this experiment are listed here: HT 8-oxo-dG ELISA Kit II(R&D Systems China Co, Ltd.No.4380–192-K), APE1(ab189474, Abcam), FEN1(ab109132, Abcam), Pol Beta (ab26343, Abcam) and XRCC1(ab1838, Abcam) antibodies, anti-γ-H2AX antibody (ab2893, Abcam).

### Preparation of compounds

5-Fu was purchased from Sigma (Cat No. F6627). JPYW was formed by *Astragalus propinquus*, *Codonopsis pilosula*, *Atractylodes macrocephala*, *Angelica sinensis*, *Paeonia lactiflora*, the pericarp of *Citrus reticulata*, ginger processed *Pinellia ternata*, *Sparganium stoloniferum*, *Curcuma zedoaria*, *Salvia chinensis*, *Hedyotis diffusa*, and *Liquiritia*, purchased from the pharmaceutical department of Jiangsu Province Hospital of Traditional Chinese Medicine.

All DNA oligos used in this experiment were synthesized from GenScript. Interference RNA against FEN1 was synthesized from HanBiology. FEN1 protein was purified from our own laboratory from E.coli.

### In vivo 8-Oxo-dG BER assay

An extrachromosomal assay system using a biotin-tagged oligonucleotide DNA substrate containing an 8-oxo-dG lesion that can be repaired by LP-BER was designed. The 8-oxodG BER assay uses a double-stranded oligonucleotide DNA substrate consists of a biotinylated DNA strand that is base paired to an untagged DNA strand containing a single 8-oxo-dG lesion. The substrate DNA is transfected into BGC823 and BGC823/5-Fu cells and incubated for 2 h to allow repair of the 8-oxo-dG lesion. JPYW was added to the corresponding culture medium. The cells are then lysed and the biotinylated substrate of DNA is captured using streptavidin-coated magnetic beads. In the presence of the magnet, the strand containing the 8-oxo-dG lesion is denatured and isolated from the streptavidin-bound biotinylated strand. The isolated DNA is then quantified and subjected using a competitive 8-oxo-dG ELISA kit [14].

### Preparation of cell extracts

Cells were incubated overnight to reach a mid-exponential growth phase, washed three times with phosphate buffer saline (PBS) and resuspended at 10^6^ cells/20 ml in buffer I [10 mM Tris–HCl (pH 7.8) and 200 mM KCl]. After the addition of an equal volume of Buffer II [10 mM Tris–HCl (pH 7.8), 200 mM KCl, 2 mM EDTA, 40% glycerol, 0.2% NP-40, 2 mM dithiothreitol, 0.5 mM phenylmethylsulfonyl fluoride (PMSF) and protease inhibitor cocktail (Roche)], the cell suspension was rocked (1 h, 48 °C) and centrifuged (10 min, 16,000 g). Extract the supernatant and store at − 80 °C. Protein concentrations were determined by Bradford protein assay.

### Reconstituted base excision repair assay

A DNA double strand with one missing site (G guanine base) was prepared in vitro as a DNA substrate. Complete repair reactions were carried out in a 20 ml reaction buffer system [40 mM HEPES–KOH (pH 7.8), 70 mM KCI, 7 mM MgCl2, 1 mM dithiothreitol, 0.5 mM EDTA, 2 mM ATP, 50 mM each of dATP, dTTP and dGTP, and 8 mM 2 mCi (a-32P)-dCTPFor]. For cell extract reconstitution, DNA substrate was incubated with the total cell extracts (5 μg). Reactions (30 min, 37 C) were stopped by adding an equal volume of Formamide-containing gel loading buffer. Then the products were resolved on 15% polyacrylamide gels containing 8 M urea and visualized by autoradiograph y[15, 16].

### Q-PCR

Cells were collected for quantitative real-time polymerase (Q-PCR) detection. Primer sequences were as follows: APE1-f:(CAAGATCTGCTCTTGGAATG);

APE1-r (AGGCAGTGGATAGCAAGCTA);

FEN1-f (TGGAGCGAGCCAAATGAA);

FEN1-r (GCCGGTCACCTTGAAGAAA);

Pol Beta-f (AGCAAGCAGCTACAATGCAA);

Pol Beta-r (AGGTGTGTACAATGTTGACTTGG);

XRCC1-f (GCCAGGGCCCCTCCTTCAA);

XRCC1-r (TACCCTCAGACCCACGAGT).

### Western blot

BGC823 and BGC823/5-Fu cells were cultured routinely or cultured with JPYW, cell proteins were extracted, and the protein concentrations were determined by Bradford protein assay. The expression of APE1, FEN1, Pol Beta and XRCC1 in each group was detected by Western blot method.

### CCK8 assay

Cells were seeded at 4000 per well in a 96-well strip plate. The cells were incubated for 48 h at different concentrations of 5-Fu. Cell™ Counting Kit-8 (CCK8, E1CK-000208, EnoGene) was added to each well, and the cells were further incubated for 4 h at 37 °C before the measurement of the absorbance at 450 nm. The survival rate was calculated at different concentrations of 5-Fu.

### Immunofluorescence staining

Cells were cultured in six-well plates containing acid-treated glass slides and were incubated overnight. The glass slides were then washed with PBS, fixed with 4% formaldehyde in PBS for 30 min, and washed with PBS again. Triton X-100 (0.05%) was added for 5 min to permeabilize the cells. The glass slides were blocked with 3% BSA and then incubated with primary antibody. The slides were washed, incubated with secondary antibody conjugated with fluorescein isothiocyanate (FITC), washed again with PBS, and stained with 4′,6-diamidino-2-phenylindole (DAPI). Then the mounted slides were viewed with a Nikon 80I 10-1500X microscope, and images were captured with a camera [17, 18].

### FEN1 nuclease activity assay

FEN1 is a structurally specific branching endonuclease. The DNA is cleaved at the junction of the Flap structure with the double-stranded DNA. At this point, the Flap structure is cut. If the cut Flap is labeled with a companion, the amount of Flap cut can be determined by autoradiography to quantify the FEN1 activity. The DNA substrates were synthesized in vitro and the isotopes ^32^P were labeled at the end of the Flap structure. ^32^P-labeled DNA substrates were incubated with purified FEN1 in a buffer solution containing 50 mM Tris-HCl (pH 8.0), 50 mM NaCl, and 5 mM MgCl2. The reactions were carried out at 37 °C for 40 min and were terminated with the stop buffer (95% formamide, 20 mM EDTA, 0.05% bromophenol blue, 0.05% xylene cyanol). The products and substrates were then separated by 15% denaturing polyacrylamide gel electrophoresis and visualized by autoradiograph y[19, 20].

### Statistical analysis

SPSS 25.0 software was used for statistical analysis. Data was presented as mean ± SD. One-way ANOVA was used for the comparison between multiple groups, and independent-samples T test was used for the comparison between two groups. *P-values < 0.05, **P < 0.01, or ***P < 0.001 were considered as statistically significant. All experiments were repeated at least three times to confirm reproducibility.

## Resullts

### BGC823/5-Fu had higher DNA repair efficiency than BGC823, both intracellularly and extracellularly

**Fig. 1:**
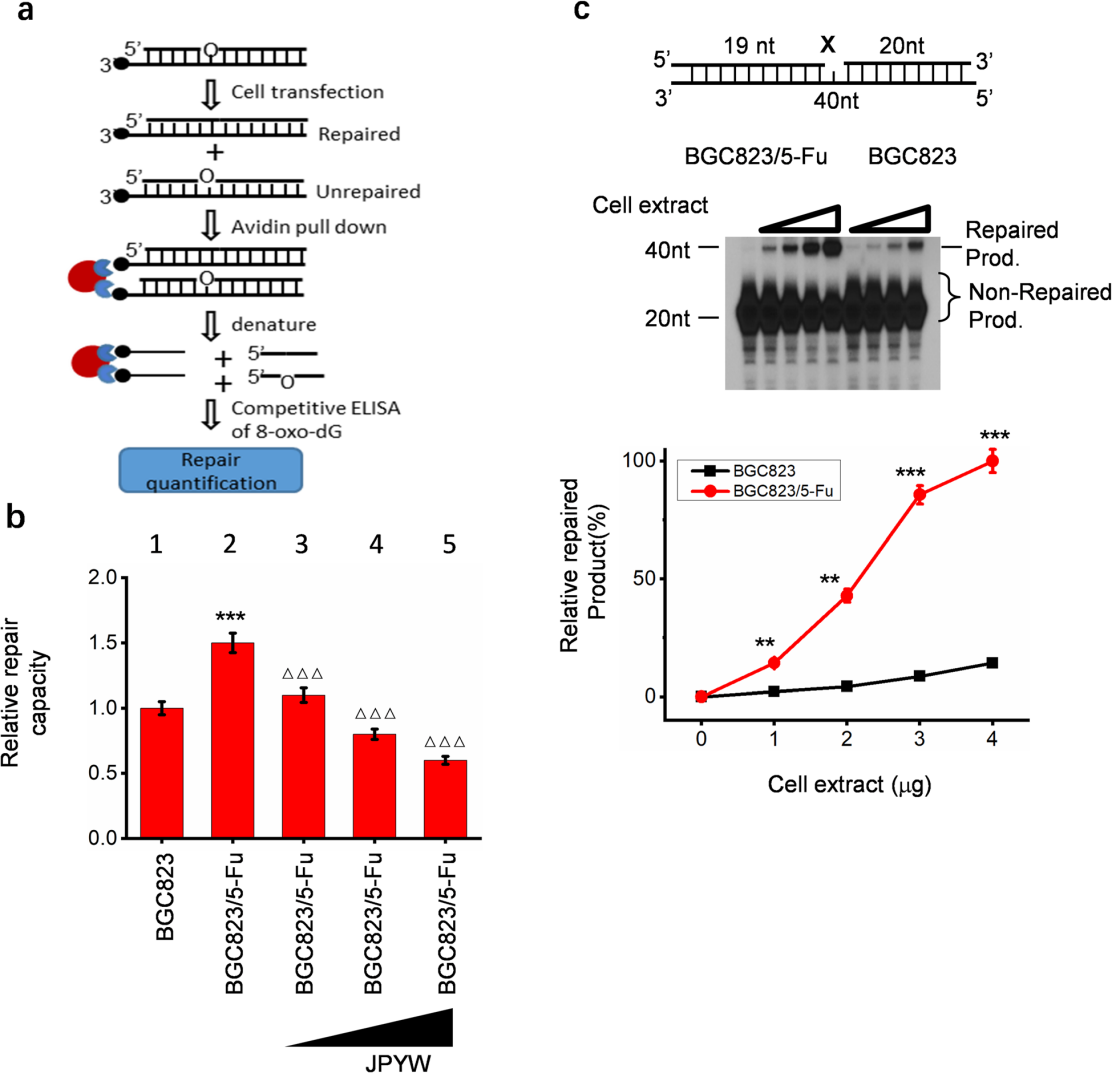
The comparison of DNA repair ability of BGC823 and BGC823/5-Fu. **a** Procedure of LP-BER assay in cells. A DNA oligo containing the damaged DNA lesion 8-oxo-dG was transfected into BGC823 and BGC823/5-Fu cells; 2 h later, cells were lysed, and released 8-oxo-dG was determined by ELISA. **b** Relative repair capacity of two cells. **c** Reconstitution of long-patch (LP) base excision repair (BER) with cell lysate . The top part showed the schematic structure of the corresponding DNA substrates. The middle showed PAGE-separated products and the bottom showed the relative percentage of repaired product at different cell extract concentrations

To compare the relative repair capacity of BGC823 and BGC823/5-Fu cells intracellularly, we adopted an extrachromosomal assay system using a biotin-tagged oligonucleotide DNA substrate containing an 8-oxo-dG lesion that can be repaired by LP-BER. The substrate DNA was transfected into BGC823 or BGC823/5-Fu cells. Incubated 2 h to allow cells to repair the lesions. Then cells were lysed to obtain DNA and the remaining 8-oxo-dG lesions were detected using a competitive 8-oxo dG ELISA Kit (Fig. 1a for assay procedure [5]). The total amount of 8-oxo-dG initially introduced into the cells was 10 ng, the amount of residual 8-oxo-dG was measured and the relative repair rate was calculated. The results suggested that BGC823/5-Fu had higher DNA repair efficiency than BGC823 intracellularly (Fig. 1b Lane1 vs Lane2).

To compare the overall LP-BER efficiency of BGC823 and BGC823/5-Fu extracellularly, we performed an in vitro LP-BER reconstitution assay using cell extracts (Fig. 1c). According to the results of autoradiography, in the reaction system of two cell lysates, as the concentration of cell lysate increased, the repair products increased in two cells, as demonstrated by the intense 40-nt bands, BGC823/5-Fu increased significantly higher than BGC823; the unrepaired products decreased, as demonstrated by the intense 20-nt bands, and BGC823/5-Fu decreased more than BGC823. Quantitative analysis was performed using Image J, and the average value was calculated to analyze the repair rate. As shown in the bottom part, the repair rate of BGC823/5-Fu was significantly higher than that of BGC823.

### FEN1 was highly expressed in BGC823/5-Fu cells

**Fig. 2:**
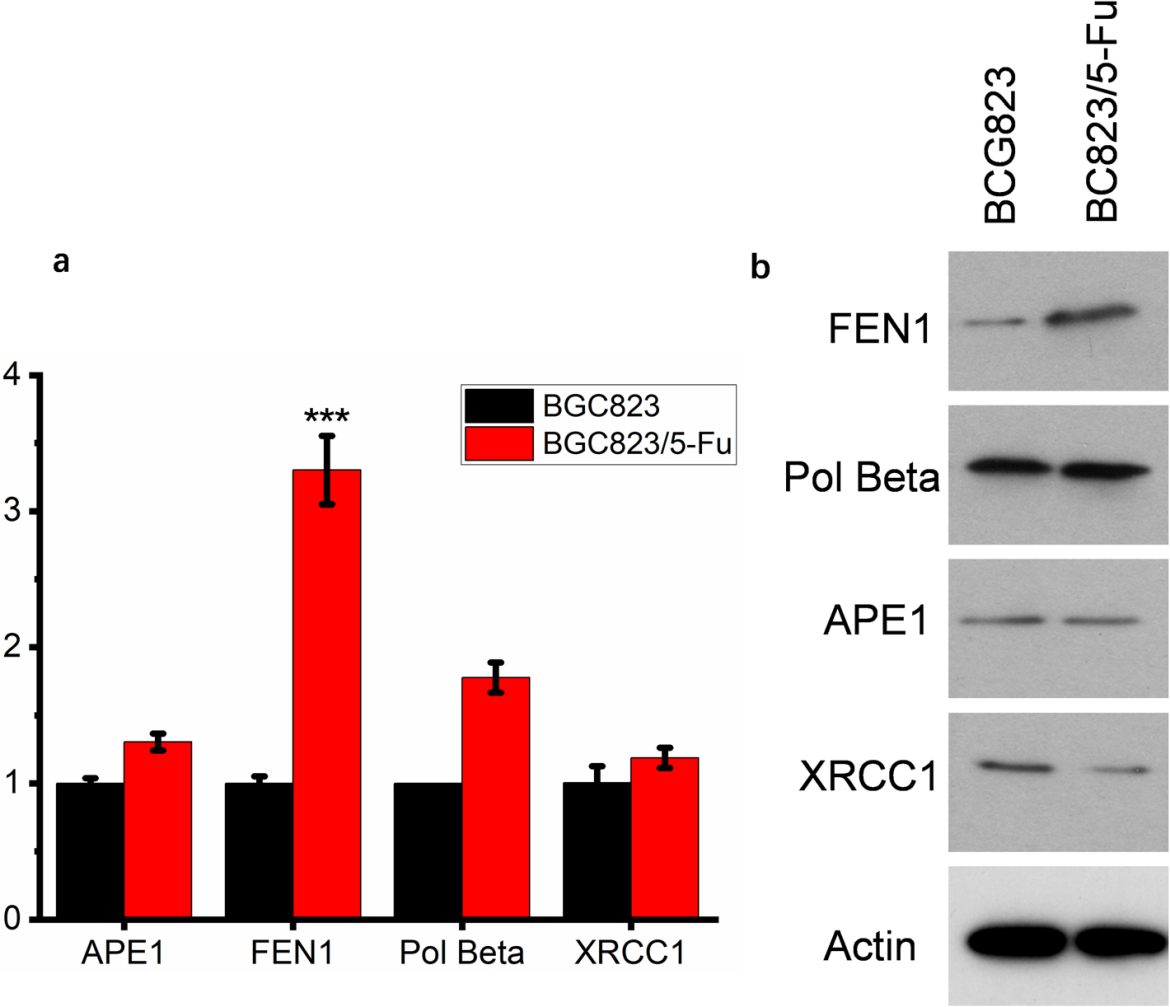
The expression of FEN1 in BGC823/5-Fu compared with BGC823. FEN1 was highly expressed cells at both (**a**) gene expression level and (**b**) protein expression level

Based on the previous results, we concluded that BGC823/5-Fu had higher DNA repair efficiency than BGC823 in both intracellular and extracellular systems, so we further explored what components in BER repair system leading to this change. LP-BER was mainly responsible for 5-Fu damage repair in cells, so the expression levels of LP-BER related DNA repair enzymes in BGC823 and BGC823/5-Fu cells were compared. At the gene level, mRNA expression levels of APE1, FEN1, Pol Beta and XRCC1 in two cells were measured by Q-PCR, the relative expression of FEN1 was significantly increased in BGC823/5-Fu (Fig. 2a). At the protein level, Western blot was used to measure the expression levels of above DNA repair enzymes. Similarly, FEN1 expression was significantly increased in BGC823/5-Fu (Fig. 2b). To sum up, it can be concluded that FEN1 is highly expressed in BGC823/5-Fu regardless of gene expression level or protein expression level.

### The expression level of FEN1 was directly related to the drug resistance of BGC823 to 5-Fu

**Fig. 3:**
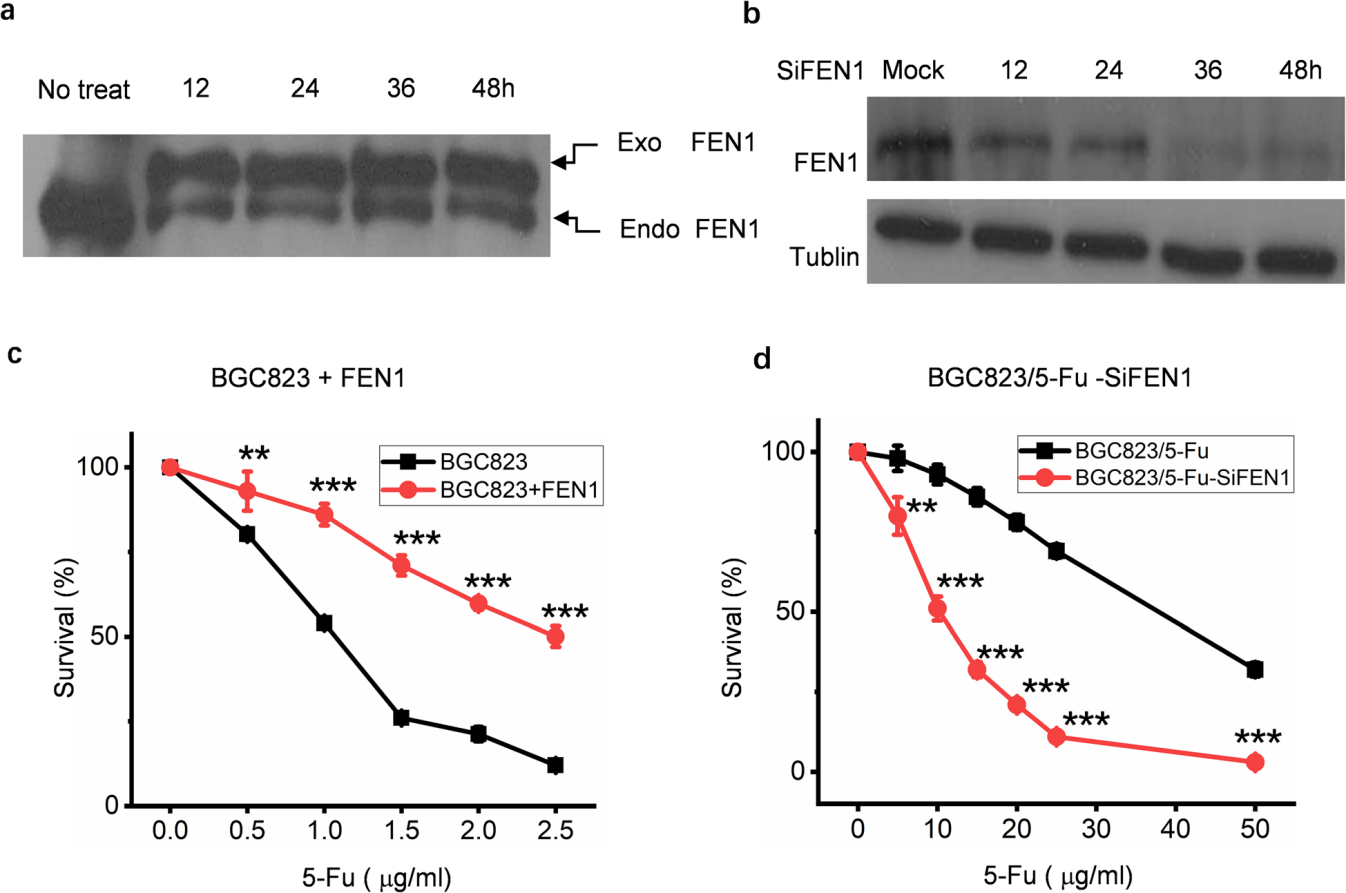
Effects of FEN1 expression on BGC823’s resistance to 5-Fu. FEN1 was (**a**) overexpressed in BGC823(the overexpression vector was pIRESneo2-FEN1–3 x C-myc) or (**b**) knocked-down in BGC823/5-Fu cells. The expression level of FEN1 was determined by Western blot analysis using anti-FEN1 antibody. FEN1 over expression in BGC823 cells caused increased resistance to 5-Fu (**c**). FEN1 knockdown sensitized BGC823/5-Fu cells to 5-Fu (**d**)

As FEN1 was involved in DNA repair and was overexpressed in the drug resistant cells, we hypothesized that manipulating FEN1 may alter the response of cancer cells to chemotherapeutic drugs that induce DNA damage. To test this, FEN1 level was manipulated in cells and the survival rate was measured after exposed to 5-Fu. FEN1 was overexpressed in BGC823 (high endogenous FEN1 expression, Fig. 3a) or suppressed in BGC823/5-Fu (low endogenous FEN1 expression, Fig. 3b). It was found that FEN1 overexpressed cells (BGC823 + FEN1) demonstrated higher resistance to 5-Fu (Fig. 3c), whereas FEN1 suppressed cells (BGC823/5-Fu-SiFEN1) showed higher sensitivity to 5-Fu (Fig. 3d) when compared to their own control groups. In summary, we confirmed that FEN1 expression level was directly related to BGC823’s resistance to 5-Fu, and it was a key gene in the 5-Fu resistance of gastric cancer cells relevant to DNA damage repair.

### JPYW inhibited DNA damage repair, both intracellular and extracellular

**Fig. 4:**
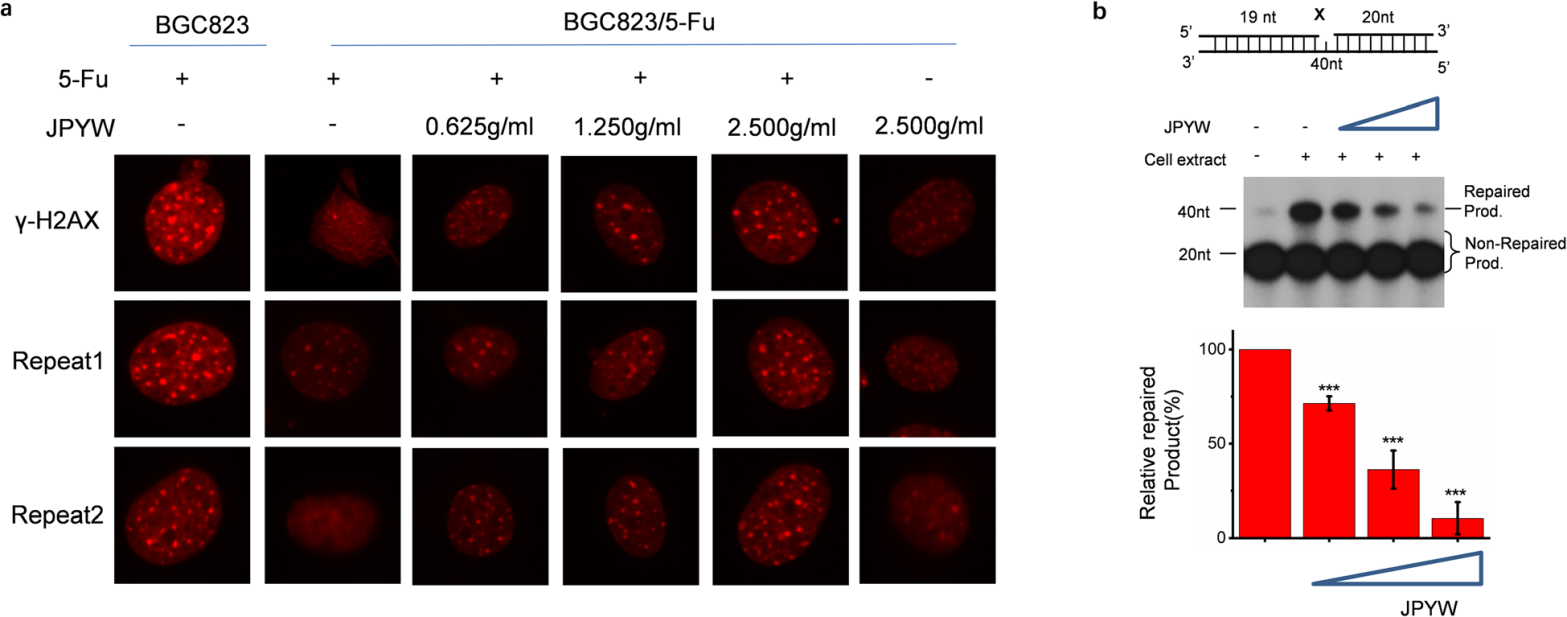
The influences of JPYW on DNA damage and DNA repair ability. **a** DNA damage in two cells treated with 5-Fu and JPYW. **b** Effect of JPYW on DNA repair ability in vitro. The top part showed the schematic structure of the corresponding DNA substrates. The middle showed PAGE-separated products and the bottom showed the relative percentage of repaired product at different JPYW concentrations

Previous reports indicated that defection in the Okazaki fragment maturation process during DNA replication or ligation during DNA repair can lead to the accumulation of DNA double strand breaks (DSBs) [21]. Therefore, we assumed that JPYW treated cells can display a higher level of DSBs. To test this, we cultured BGC823 and BGC823/5-Fu cells with 5-Fu (5μg/ml), JPYW (0.625 g/ml, 1.250 g/ml, 2.500 g/ml), followed by immunofluorescence staining to detect γ-H2AX foci in two cells using anti-γ-H2AX. The phosphorylation of γ-H2AX increased with more DNA damage in the cells. Under a fluorescence microscope, the brighter spots there were, the more damage was labeled inside the cells. In BGC823/5-Fu cells, when 5-Fu added in company with JPYW, the fluorescence microscope image showed a large number of bright highlights and it was JPYW concentration dependent. However, only adding JPYW without 5-Fu showed rare DNA damage, suggesting that JPYW synergistically caused DNA damage along with 5-Fu (Fig. 4a). It revealed that JPYW directly inhibited the DNA damage repair of BGC823/5-Fu intracellularly.

To further explore the effect of JPYW on DNA damage repair ability in cells, an extrachromosomal assay system using a biotin-tagged oligonucleotide DNA substrate containing an 8-oxo-dG lesion was adopted. As shown in Fig. 1b, JPYW treated BGC823/5-Fu cells showed significantly lower repair ability than the untreated BGC823/5-Fu cells. Once upon addition of JPYW, a decrease in 8-oxo dG repair was observed at a dose dependent manner, suggesting that JPYW inhibited endogenous BER at the intracellular level (Fig. 1b, Lane 3,4,5 vs Lane2).

At the extracellular level, the overall LP-BER efficiency of JPYW was detected in vitro LP-BER reconstitution assay using cell extracts (Fig. 4b). According to the results of autoradiography, in the reaction system of cell lysates and JPYW, as the concentration of JPYW increased (0.625 g/ml, 1.250 g/ml, 2.500 g/ml), the repair products decreased in BGC823/5-Fu, as demonstrated by the intense 40-nt bands. Quantitative analysis was performed using Image J, and the average value was calculated to analyze the relative repair rate. As shown in the bottom figure, with the increasing concentration of JPYW, the repair ability of cell lysates decreased, indicating that JPYW inhibited BER activity extracellularly.

### JPYW directly inhibited FEN1 expression in BGC823/5-Fu

**Fig. 5:**
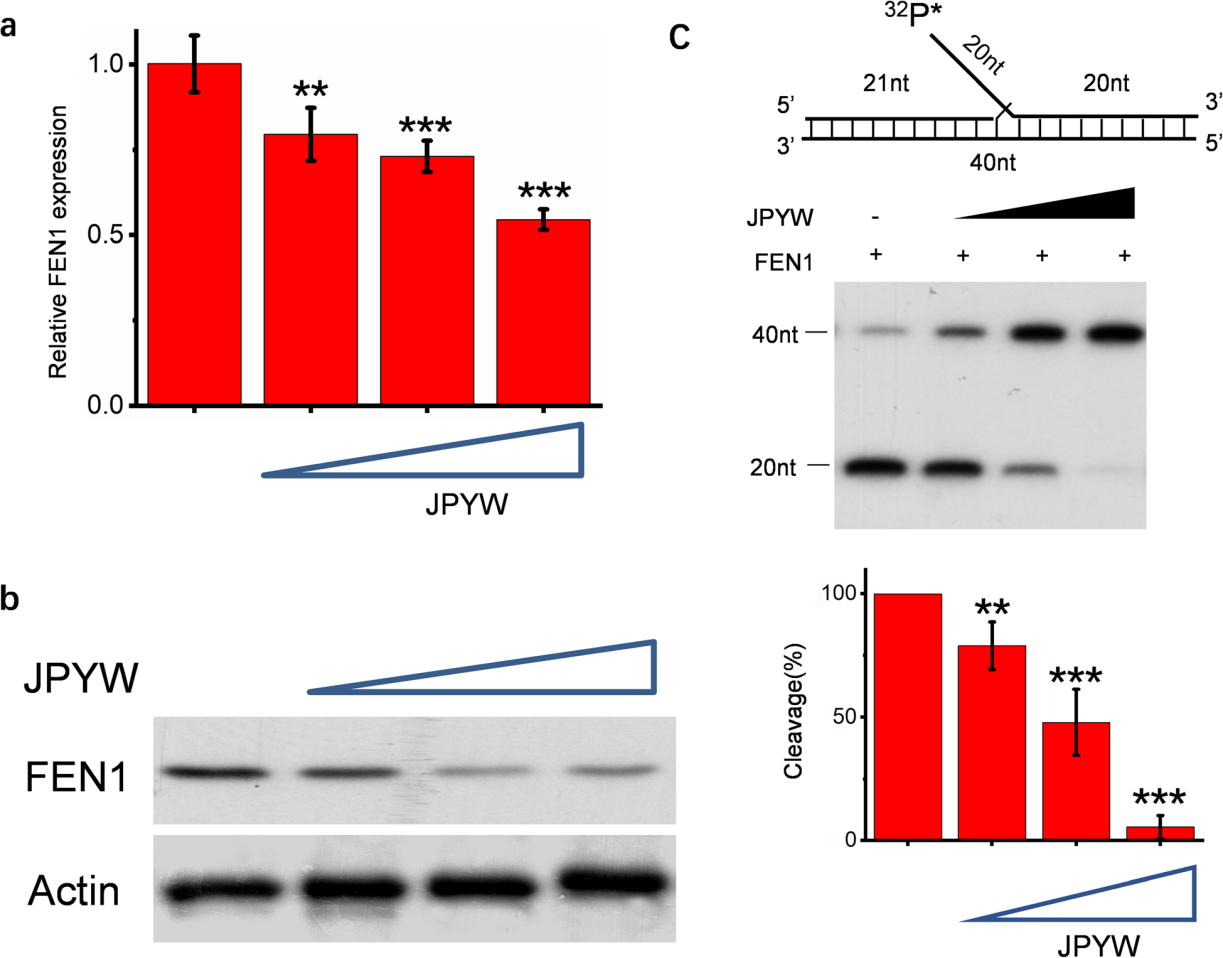
Effects of JPYW on FEN1 expression and cleavage ability. JPYW directly inhibited FEN1 expression of BGC823/5-Fu cells at both (**a**) gene level and (**b**) protein level. **c** JPYW inhibited the activity of FEN1 at the enzymatic level .The top part showed the synthetic 32p labeled DNA substrates. The middle showed PAGE-separated products and the bottom showed the cleavage percentage at different JPYW concentrations

According to the above researches, we found that JPYW can indeed inhibit DNA damage repair both intracellularly and extracellularly. As FEN1 was highly expressed in BGC823/5-Fu cells, we were wondering if it’s possible that it achieved this effect by acting on FEN1. We cultured BGC823/5-Fu cells with different JPYW concentrations (0.625 g/ml, 1.250 g/ml, 2.500 g/ml), and used Q-PCR to detecte the mRNA expression levels of FEN1. As shown in Fig. 5a, with the concentration of JPYW increased, the relative FEN1 expression decreased gradually. Western blot was used to measure the protein expression of FEN1 and the results was similar (Fig. 5b). Therefore, it can be concluded that FEN1 expression in BGC823/5-Fu decreased with the increased concentration of JPYW regardless of gene expression level or protein expression level, indicating that JPYW directly inhibited FEN1 expression in BGC823/5-Fu.

### JPYW inhibited the activity of FEN1 at the enzymatic level

FEN1 is a structure specific branching endonuclease, and the flap structure was excised by cutting the DNA at the binding point of Flap structure and double-stranded DNA. The FEN1 substrate was synthesized in vitro and the flap was labeled with an isotope ^32^P. JPYW (0.625 g/ml, 1.250 g/ml, 2.500 g/ml) and the purified FEN1 protein (1 ng) were added and the amount of Flap excised was measured by autoradiography after the reaction. With the increased concentration of JPYW, less Flap structures labeled with ^32^p were cut off, as demonstrated by the intense 20-nt bands (Fig. 5c). Image J was used for quantitative analysis, and the average value was calculated to analyze the cleavage rate. As shown in the bottom figure, the relative cleavage rate decreased with increase in JPYW concentration. This suggested that JPYW inhibited FEN1 activity at the enzymatic level.

## Discussion

Jianpi Yangwei Decoction was created by the famous National Chinese Medicine Practitioner Professor Shenlin Liu through summing up decades of experience on diagnosis and treatment of gastric cancer. The curative effect of this prescription has been proved by clinical observations and basic experiment studies, especially in combination with chemotherapy. In the clinical research, through the State Administration of Traditional Chinese Medicine multi-center clinical research program (NO.200807022), taking 8 years to follow up 489 cases, the research results demonstrated that the prescription had the effect of decreasing the recurrence rate of patients with stage II and III gastric cancer. The risk of recurrence was decreased by 32.8% compared with chemotherapy alone (*P* = 0.0042, ^9]^. In basic experiment studies, our team constructed a 5-Fu-resistant gastric cancer cell line BGC823/5-Fu. Through in vivo and in vitro experiment studies, it was proved that Jianpi Yangwei Decoction can inhibit the PI3K/AKT signaling pathway of drug-resistant cells and down regulate the expression of drug-resistant proteins. Thereby, the resistance of gastric cancer cells to 5-Fu was reversed [10, 13]. However, it is well known that traditional Chinese medicine compound has the biological characteristics of multiple targets and multiple pathways. So, it is necessary to investigate its drug resistance reversal mechanism from different angles and aspects.

The drug resistance mechanisms of gastric cancer found in previous studies include: increased drug efflux and metabolic abnormalities, increased DNA damage repair and reduced apoptosis, modification or alteration of drug active target proteins, the presence of tumor stem cells, and the transformation of epithelial cells to mesenchymal cells, regulation of hypoxia and hypoxia-inducible factor-1α and microRNAs, et al .[22] Among them, DNA damage repair is a new and important mechanism of tumor drug resistance and it is also a hot-topic at present. Recent studies have shown that the BER activity as an important pathway of DNA damage repair is related to the chemotherapy sensitivity, and the studies of FEN1 have been widely concerne d[23–25]. Multiple studies have demonstrated that high FEN1 expression is associated with drug resistance in a variety of malignancies. In both breast cancer [5] and lung cancer [6] drug-resistant cells, the level of FEN1 medicated DNA damage repair was significantly increased and the inhibition of FEN1 expression could promoted the drug-resistant cells sensitivity to Tamoxifen and Cisplatin respectively. The same situation was true with cervical cancer. Cervical cancer highly expressed FEN1, and inhibiting FEN1 expression can make cervical cancer cells sensitive to Paclitaxe l[7]. In osteosarcoma, [8] mir-193b /FEN1 axis increased the sensitivity of osteosarcoma cells to Epirubicin by inducing autophagy and apoptosis.

In the meantime, existing studies have shown that FEN1 is highly expressed in gastric cancer, which is closely related to the prognosis of gastric cancer. It is one of the potential tumor markers for gastric cancer, and reducing the FEN1 expression can effectively improve the chemotherapy sensitivity of gastric cancer. Wang K [26] discovered that about 76% human gastric cancer tissues had higher FEN1 expression in comparison to the corresponding normal gastric tissues. Moreover, FEN1 expression had a positive correlation with the degree of differentiation, lymphatic metastasis, tumor TNM stage of gastric cancer. Xie C [27] found that FEN1 was significantly induced by CDDP in a dose and time dependent manner, while the inhibition of FEN1 could effectively reduce the proliferation and increased the apoptotic rate of SGC7901. In the cells disposed with FEN1-siRNA and exposed to CDDP, the expressions of apoptosis related proteins Bcl-2 and Bcl-xl were restrained, and the expression of Bax was promoted. The study indicated that inhibiting FEN1 was one of the effective ways to improve the gastric cancer’s sensitivity to chemotherapy.

As 5-Fu is a cell cycle specific drug, it has a killing effect on tumor cells at the stage of DNA synthesis. Based on the above research conclusions, it is not difficult to speculate that the resistance of gastric cancer to 5-Fu may also be related to FEN1 mediated DNA damage repair. Therefore, it is of great significance to seek out a drug resistance reversal agent for DNA damage repair pathways associated with 5-Fu resistance in gastric cancer in order to inhibit drug resistance and improve the clinical treatment efficacy.

In recent years, studies on DNA damage repair mediated drug resistance reversal agents have focused on DNA repair inhibitors. Among them, studies on western drug reversal agents mainly include: PARP inhibitor Olaparib targeting DNA damage repair gene of BRCA1/2, [28] DNA-PK inhibitor CC-115, [29, 30] ATR inhibitor VX-970 and M6620, [31, 32] CHK1 inhibitor LY2603618, [33] WEE1 inhibitor AZD177 5 [34] and FEN1 inhibitor SC13 [5, 35]. Traditional Chinese medicine monomer reversal agents have also been reported. Curcumin, [36] as an inhibitor of FA/BRCA pathway, can inhibit the DNA damage repair function of FA/BRCA pathway in lung cancer cells and reverse the resistance of lung cancer cells to Cisplatin. *Radix Angelicae Sinensis* and *Radix Hedysari* ultrafiltration [37] can increase the radiation sensitivity of HepG2 and H22 cells to 12C6^+^ bundles, down regulate the expression of important factors RAD51 and Ku70 in DNA repair, inhibit the repair of γ-H2AX protein, and increase apoptosis. Artesunate [38] can aggravate DNA damage of esophageal cancer cells and prolong the formation of γ-H2AX foci induced by irradiation. Artesunate induced radiosensitivity of TE-1 cells in vitro and in vivo, and was a promising radiosensitizer for esophageal cancer treatment. However, most drugs are still in the stage of new drug development or clinical trials, and the long term effect of use on patients is still unclear. While traditional Chinese medicine monomers cannot meet the requirements of TCM clinical syndrome differentiation for treatment. So, the clinical application is difficult to be widely carried out. Therefore, the study of multi-target and low-toxicity Chinese medicine compound reversal agent has been put on the agenda.

In response to this hot research area and the current limitations of DNA damage repair mediated drug resistance reversal agents, in this study, we explored the effect of Chinese herbal compound Jianpi Yangwei Decoction reversing gastric cancer’s drug resisatance to 5-Fu by targeting FEN1 to inhibit DNA damage repair. The results showed that Jianpi Yangwei Decoction inhibited DNA damage repair of gastric cancer by reducing FEN1 expression to reverse 5-Fu resistance to gastric cancer. In addition, it was found that Jianpi Yangwei Decoction not only directly inhibited the expression of FEN1 in BGC823/5-Fu, but also inhibited the functional activity of FEN1 from the enzymatic level. This indicated that the role of this prescription in DNA damage repair was also multi-faceted. The expression mechanism of Fen1 gene is regulated by multiple pathways rather than a single one, which leading the direction for future studies.

## Conclusion

In conclusion, FEN1 was highly expressed in drug resistance gastric cancer cells BGC823/5-Fu, leading to the increase of DNA damege repair level and the reduction of 5-Fu chemotherapy sensitivity, which made it an important factor in drug resistance of gastric cancer. This was a new discovery following the association of FEN1 with drug resistance to breast cancer, [5] lung cancer, [6] cervical cancer [7] and osteosarcoma [8]. Jianpi Yangwei Decoction reduced FEN1 expression level and inhibited FEN1 cleavage ability to cooperated with 5-Fu increasing DNA damage in BGC823/5-Fu, which made it an effective 5-Fu drug resistance reverser based on FEN1 mediated DNA damage repair. In future, more researches are needed to confirm its application and research value in drug resistance of gastric cancer.

## Data Availability

The datasets used and/or analyzed during the current study available from the corresponding author on reasonable request.

## Abbreviations

FEN1: Flap Endonuclease 1
JPYW: Jianpi Yangwei decoction
TCM: Traditional Chinese Medicine
5-Fu: 5-Fluorouracil
MDR: multi-drug resistance
BER: DNA Base Excision Repai
APE1: Apurinic/apyrimidinic endonuclease 1
Pol Beta: DNA polymerase beta
XRCC1: X-ray Repair complementing defective repair in Chinese hamster cells 1
LP-BER: Long patch BER pathway
PBS: phosphate buffer saline
EDTA: Ethylenedinitrilotetraacetic acid
PMSF: Phenylmethylsulfonyl fluoride
Q-PCR: quantitative real-time polymerase
BSA: Albumin from bovine serum
FITC: Fluorescein isothiocyanate
DAPI: 4′,6-diamidino-2-phenylindole
DSBs: DNA double strand breaks
